# An Exploratory Case Control Study of Risk Factors for Hepatitis E in Rural Bangladesh

**DOI:** 10.1371/journal.pone.0061351

**Published:** 2013-05-13

**Authors:** Alain B. Labrique, K. Zaman, Zahid Hossain, Parimalendu Saha, Mohammad Yunus, Anowar Hossain, John Ticehurst, Brittany Kmush, Kenrad E. Nelson

**Affiliations:** 1 Department of International Health, Johns Hopkins Bloomberg School of Public Health, Baltimore, Maryland, United States of America; 2 International Center for Diarrheal Disease Research, Bangladesh (icddr,b), Dhaka, Bangladesh; 3 Department of Pathology, Johns Hopkins School of Medicine, Baltimore, Maryland, United States of America; 4 Department of Epidemiology, Johns Hopkins Bloomberg School of Public Health, Baltimore, Maryland, United States of America; University of Iowa, United States of America

## Abstract

Hepatitis E virus (HEV) is the major cause of epidemic and sporadic hepatitis globally. Outbreaks are associated with fecal contamination of drinking water, yet the environmental reservoir of HEV between epidemics remains unclear. In contrast to neighboring countries, where epidemics and sporadic disease co-occur, HEV-endemic communities in rural Bangladesh seldom report outbreaks; sporadic hepatitis E is reported from urban and rural areas of the country. Besides typical enteric risk factors, other routes for HEV infection and disease are unclear. We conducted monthly household surveillance of a southern Bangladeshi community of 23,500 people to find incident cases of acute hepatitis E over a 22 month period. An algorithm was used to capture 279 candidate cases, of which 46 were confirmed acute HEV infections. An exploratory case-control study was conducted to identify putative risk factors for disease. Nearly 70% of cases were over 15 years old. Female gender seemed protective (OR:0.34) against hepatitis E in this conservative setting, as was the use of sanitary latrines (OR:0.28). Socioeconomic status or animal exposures were not significant predictors of disease, although outdoor employment and recent urban travel were. Unexpectedly, recent contact with a “jaundiced” patient and a history of injection exposure in the 3 months prior to disease (OR:15.50) were significant. Susceptible individuals from “endemic” communities share similar enteric exposure risks to those commonly associated with tourists from non-endemic countries. This study also raises the novel possibility of parenteral and person-to-person transmission of HEV in non-epidemic, sporadic disease settings.

## Introduction

Hepatitis E virus (HEV) is a ribonucleic acid (RNA) virus, recognized today as the leading cause of acute viral hepatitis both in epidemic form and as the cause of sporadic disease across much of the developing world, especially in countries where community sanitation is poor [1], [2]. Although initially thought to be limited in endemicity to developing countries, the geographic distribution of HEV now includes most of Europe as well and the United States, albeit mostly attributed to the largely zoonotic genotype 3, mainly circulating in swine and other wild game populations [3].

Studies of genotype 1 HEV infection have suggested that only 20–30% of infections result in clinical symptoms [4], [5]. Even then, hepatitis E disease (hepatitis E) is self-limiting and does not usually result in long-term sequelae [2], [6]. The consequences of HEV infections in pregnancy are severe, and have been associated with high case fatality rates (up to 20%), the pathogenesis of which is not entirely clear [1], [7], [8]. At least two candidate vaccines for HEV have been developed and tested in clinical trials, but are not yet available for public use [9], [10].

Over the past two decades a large number of well-documented and careful studies have been conducted of HEV epidemics and many seroepidemiologic and cross-sectional studies have assessed the prevalence of antibodies to HEV in countries from Latin America to Southeast Asia [11]–[14]. Both large-scale and local outbreaks of genotype 1 hepatitis E are often clearly preceded by a dramatic water contamination event leading to the transmission of high levels of HEV through drinking water [8], [15]. Accidental cross-contamination between sewer and water supply lines, seasonal flooding, as is common in South Asia, compounded by dilapidated drinking water supplies are commonly found to be at the source of outbreaks [16]–[19].

Corwin and colleagues summarized several epidemiologic studies conducted in southeast Asia which attempted to describe risk factors for increased HEV seroprevalence and sporadic disease [20]. These included outbreak investigations, cross-sectional prevalence studies and hospital-based case control studies. The disposal of human waste in rivers which are also used as a source for drinking, washing and cooking were found to be most significantly associated with an increased population prevalence of antibodies to HEV [20]. Other risk factors explored in the literature include livestock ownership or contact with raw animal products and/or excrement [21], [22]. Studies of HEV in travelers frequently reveal a history of visiting an endemic country, with associated high-risk enteric exposures, prior to infection [23], [24].

It remains unclear how HEV genotype 1 is maintained in a population in non-epidemic settings, and what factors predispose individuals to be infected by HEV and, subsequently, progress to disease. An exploratory nested case control study, with longitudinal community-based surveillance for acute hepatitis illness, was undertaken to identify potential risk factors for sporadic hepatitis E in a rural Bangladeshi population. The objective of the study was to compare cases of hepatitis E to healthy sero-naïve controls in an attempt to identify any clear putative risk factors (including socioeconomic status, travel, water exposures and use patterns, domestic and wild animal exposures, parenteral exposures and hygiene behaviors).

In animal models of HEV, the manifestation of clinical signs is dose-dependent and may require over a 1000-fold difference in the challenge doses compared to that sufficient for infection [25]. This observation is also supported by a low disease to infection rate [4]. If clinical disease in humans also represents the most severe exposures to sources of HEV, the proposed study hoped to draw out the most dramatic associations between “sporadic” HEV and potential risk factors for infection in a unique HEV-endemic community, from which no outbreak of hepatitis E has been documented in over 30 years of careful surveillance and study.

## Materials and Methods

Study procedures were approved by the Committee on Human Research at the Johns Hopkins Bloomberg School of Public Health and by the International Center for Diarrheal Disease Research, Bangladesh, Ethical Review Committee. Written (signature or thumbprint) informed consent was obtained from all adults participants, supported by the signature or thumbprint of a second adult witness to the consent process; parental consent was sought for children, accompanied by age-appropriate assent. Participants testing positive for antibodies to HEV, HBV, or HCV were informed of their status and counseled using ethics committee-approved messages.

### Study site and Subjects

**Figure 1:**
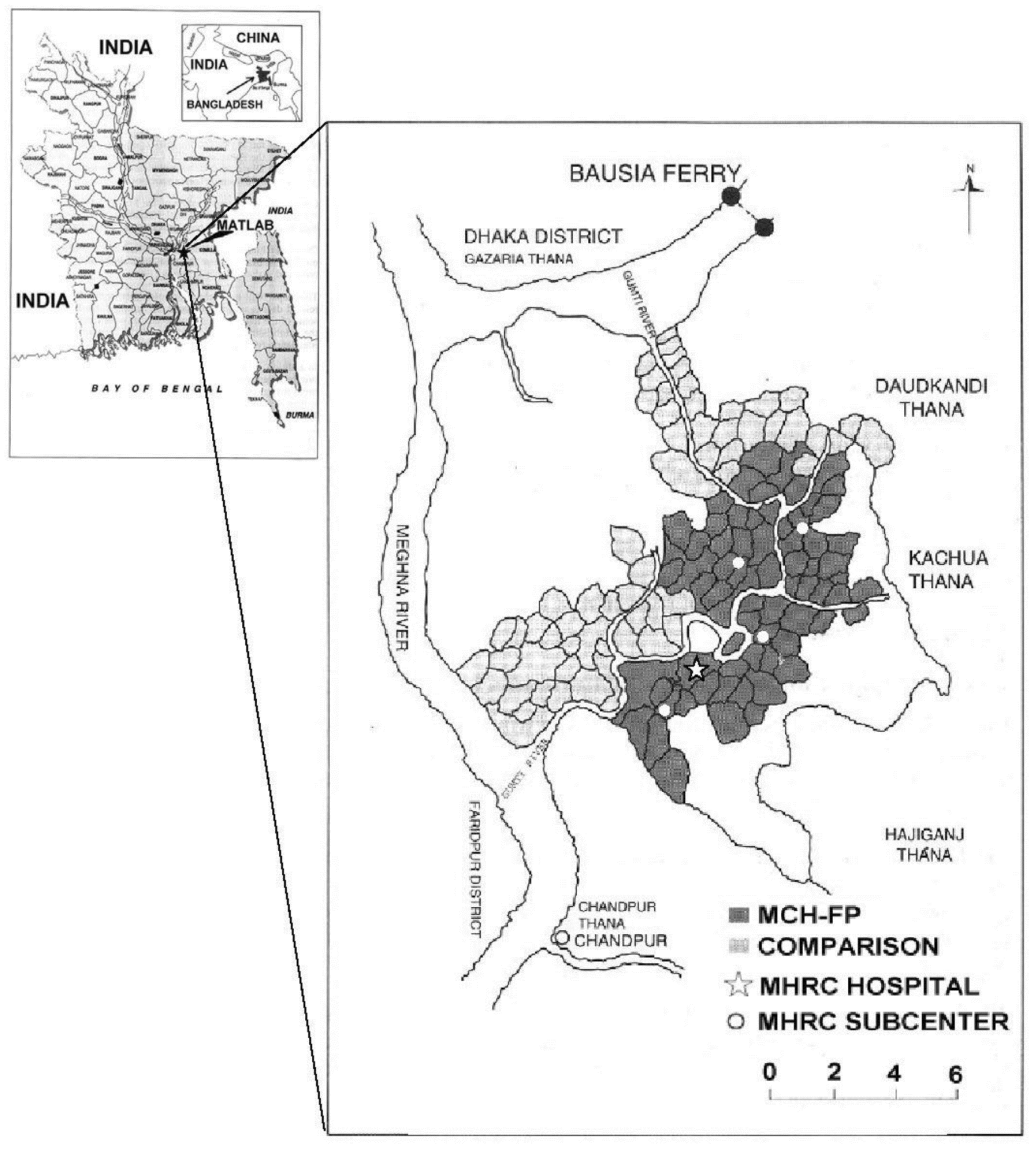
Geographic location and layout of the ICDDR,B Matlab Health Research Center. * *Modified, with permission, from Canada's International Development Research Center, www.idrc.ca and the ICDDR,B [52].

The Matlab Demographic and Health Surveillance System (MDHSS) cohort of the International Center for Diarrheal Disease Research, Bangladesh (ICDDR,B) provided an ideal setting to conduct a nested case-control study, with prospective case detection, given the existing infrastructure of a decades-old demographic and health surveillance system. The Matlab research area covers approximately 147 villages – a contiguous, rural population near 210,000 people (Figure 1) [26], [27]. Since the late 1970's, this enumerated population has participated in numerous cohort and clinical studies facilitated by a strong network of health workers connected to a central hospital and laboratory facility [28]. Although the Matlab population has been exposed to a number of public health interventions over the past decades, several statistical and demographic indicators support the generalizability of findings to rural populations in the region; these include the age structure, adult literacy, maternal mortality and total fertility rates [29]. The causes of deaths among adults in Matlab are similar to those outside the study area. However, mortality of children under five (86 per 1000 live births) is significantly lower than the rates seen at a national level (116 per 1000 live births), due to aggressive immunization strategies and active child health programs [29].

In the Matlab cohort, trained community health research workers (CHRWs) visit every household in the research area to record vital events (births and deaths) and migration once per month [30]. In the area under active demographic surveillance (figure 1),∼57 CHRWs (about 1 per 1800 people) visit between 15 to 20 households, each, per day [26].

For 22 months, a prospective surveillance system was implemented in one of the sectors of the Matlab study area to identify individuals with symptoms consistent with acute hepatitis E as “possible cases”. An entire study block of the MDHSS was selected for hepatitis surveillance based on ease of access for research teams based in the Matlab Hospital complex. (A prior HEV seroprevalence study in this population found no difference in anti-HEV seroprevalence between the four MDHSS blocks [6].) Community-based, incident cases of sporadic hepatitis E were compared to age-matched individuals from the same community without a history of symptoms or anti-HEV seropositivity.

### Developing a Surveillance Instrument

Although sign and symptom-based acute hepatitis screening has been used in hospital-based settings or by clinically-trained field workers to identify incident hepatitis cases (Fix A., Personal Communication, May 2000), we were challenged to develop an appropriate screening tool for minimally-trained community health research workers (CHRWs) to identify acute hepatitis illness.

Three rounds of month-long field testing were conducted to develop and refine a scoring method based on symptoms used in HEV studies in Egypt and Nepal [31]. These included specific symptoms of hepatitis infection such as jaundice, scleral icterus, hepatomegaly, dark urine, clay/ash colored stools, right upper quadrant (RUQ) abdominal pain or liver tenderness, as well as non-specific symptoms such as anorexia, nausea or vomiting, weakness/lassitude, malaise and fever, also commonly associated with acute hepatitis infections. A visual card (http://bit.ly/HepE_Card), depicting a patient with scleral icterus, jaundiced skin, and containing a range of color bands to verify reported “tea-colored urine” or “clay-colored stools” was developed to accompany the closed-ended scoring questionnaire used by the CHRWs [32]. The local understanding of “jaundice”, a commonly used term in this community, was investigated in case it could serve as a means of identifying candidate cases. The local terms, along with self-reported RUQ pain, were removed from the algorithm as these were found to be insensitive in identifying true hepatitis illness, and were over-reported along with other non-specific symptoms [33]. Fever and severe weakness were combined to form a single category, to help reduce non-specific reporting.

The final screening algorithm (Table 1) involved a point-based system to identify individuals most likely to be cases of acute hepatitis. In the interval since the CHRW's prior visit (30 days, on average) individuals reporting nausea or vomiting were given 1 point, fever or lassitude (such that normal work is impeded) – 1 point, anorexia (or repulsion by the smell of food, a symptom which our physician-confirmed acute hepatitis cases reported frequently) – 2 points, yellow eyes or yellow skin – 2 points, and dark (tea-colored) urine or ash/clay colored stools – 2 points. A greater number of possible points were given to categories that were likely to be more specific for acute hepatitis.

**Table 1:** Sign and symptom-based visual algorithm (http://bit.ly/HepE_Card) used to identify candidate cases of acute viral hepatitis.

Components of Illness Score	Points
Nausea *or* Vomiting	1
Fever *or* Lassitude (such that normal work is impeded)	1
Anorexia (or repulsion by smell of food)	2
Yellow Eyes *or* Yellow Skin	2
Dark Urine *or* Ash/Clay-colored stools	2

### Hepatitis Disease Surveillance

As part of several ongoing demographic and health surveillance activities, CHRWs were required to visit every household in their catchment area once every 30 days. The nested hepatitis surveillance system was activated on August 22, 2004. During her monthly visit to a household, the CHRW identified a key adult respondent whom she would ask whether any member of his/her household had experienced any of the five symptoms described above in the past 30 days. When asking about yellow eyes or skin and dark urine or clay-colored stools, the CHRW followed up any positive response with a visual probe using the card [32]. Any non-zero score was recorded in the surveillance logbook, and the total score was calculated for each person with at least one morbidity. Individuals under 1 year of age were not eligible for enrollment into the study due to cultural restrictions on blood drawing as well as the limited value of data from that demographic for this exploratory risk factor study.

### Candidate cases

The objective of the surveillance was to serve as a first-stage screening to identify acutely ill individuals with hepatitis-like signs and symptoms for eventual anti-HEV confirmation. Over 22 months, in response to a lower-than-expected candidate caseload, the criteria were adjusted to increase the number of candidate cases identified. From rounds 1 to 7, individuals with an illness score of 6, with symptoms onset within 14 days was selected for antibody screening. From round 8 to 11, the illness score cutoff was lowered to 5, and finally, from rounds 12 to 22, the window of time since the onset of symptoms was increased to 30 days, keeping the score cutoff at 5.

Upon identification of a candidate case, a notification card containing the subject's identifiers, illness score and duration since symptom onset was completed. A two-member investigation team comprising an interviewer and a phlebotomist was dispatched to the household within 48 hours. After reviewing the symptoms to confirm the illness score and onset, the candidate case was pre-enrolled through an informed consent process.

### Case Pre-enrollment and Confirmation

**Figure 2:**
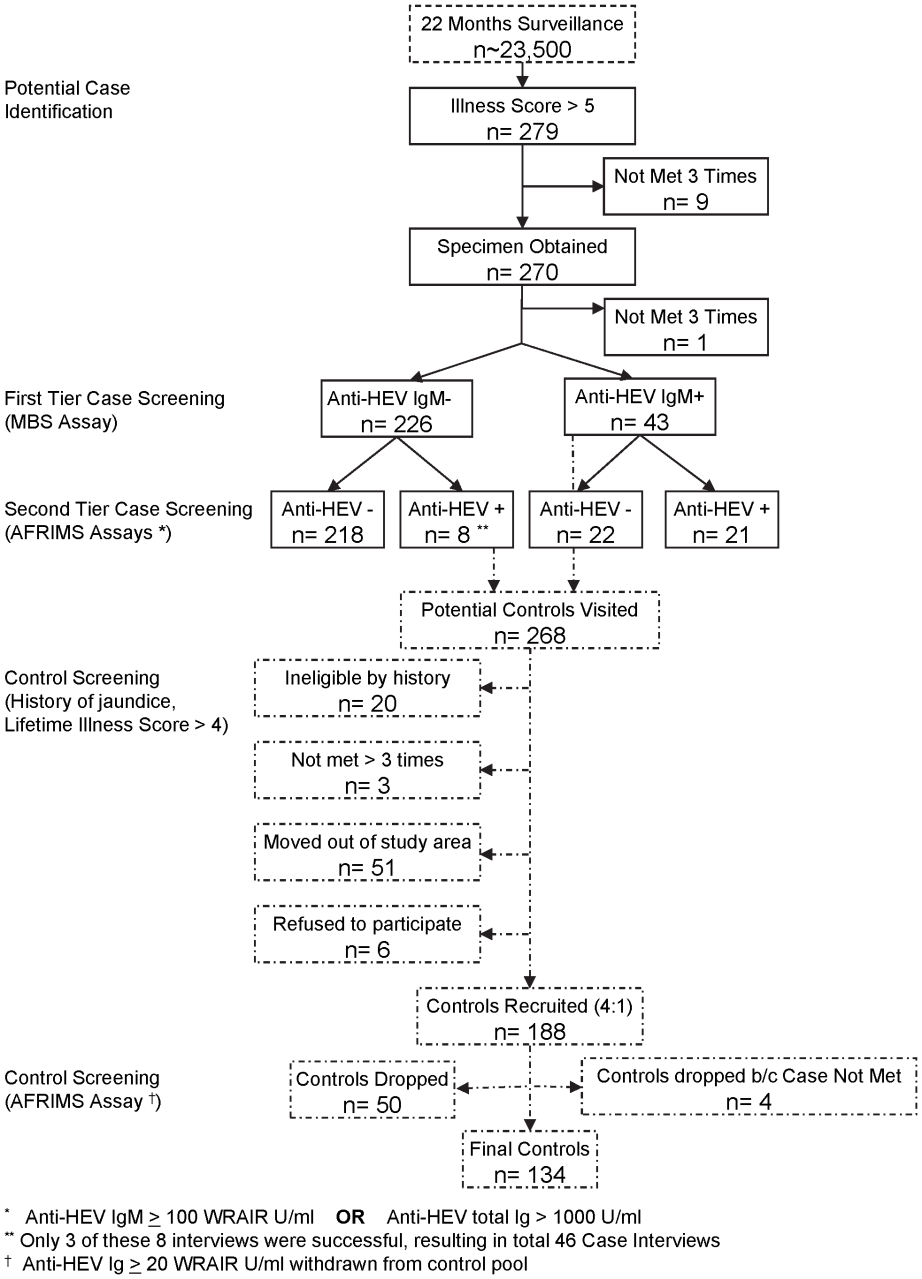
Case and control screening and selection diagram.

Pre-enrollment involved the drawing, by fingerstick, a small amount of blood as described below. This specimen was processed in the Matlab hospital laboratory, and a 30 ul aliquot of the supernatant serum was transferred on ice to the Molecular and Serodiagnostic Unit, Laboratory Sciences Division, ICDDR,B (Dhaka) for confirmatory testing by commercial enzyme immunoassay (EIA) for anti-HEV immunoglobulin (Ig) M (Molecular Biological Service-M.B.S. S.R.L., Italy) and anti-HAV IgM (DiaSorine, Italy) for individuals aged less than 10. This first-tier case screening is described in more detail below, under Laboratory Analysis (Figure 2).

If the first-tier anti-HEV IgM result was positive, the individual was subjected to a detailed exposure and history questionnaire. Confirmed cases were only defined as those who, after identification as acutely ill and seropositive by commercial immunoassay, had a second positive anti-HEV result from the Virology Laboratory of the Armed Forces Research Institute of Medical Sciences (AFRIMS), a regional reference laboratory for viral hepatitis in Bangkok, Thailand (see Figure 2). An AFRIMS anti-HEV IgM titer≥100 (Walter Reed Army Institute of Research units per milliliter (WRAIR U/ml), or an AFRIMS anti-HEV total Ig titer≥1000 U/ml was considered confirmation of an acute HEV infection. Any cases identified as acute infections by the AFRIMS assay but missed by the first-tier commercial assay were re-visited by the study team, who attempted to enroll them into the study.

### Control Selection

We aimed, in this exploratory study, to identify controls with no prior hepatitis E virus infection and thus, disease as the most extreme comparator for the case group. For each first-tier confirmed case, four age-matched *potential* controls were selected from the Matlab HDSS population database immediately after the case was confirmed (incidence-density sampling). A list of eight possible age-matches were visited in the order selected, until four were successfully recruited. We anticipated, based on known anti-HEV seroprevalence from this population [34], that the cases would likely be older than randomly selected controls from the population. Matching also controlled for age as a potential confounder, using year of birth as a precise matching criterion.

Controls were visited and, if consenting to participate, screened using a brief interview. Any control reporting having prior jaundice or hepatitis illness was disqualified from enrollment. Furthermore, any history of illness consistent with acute viral hepatitis (using a similar scoring system to the Illness Score) resulted in disqualification; this screening was done to maximize the chance of capturing true seronaïve controls. Each control was interviewed with the same assessment instrument used for cases. A small fingerstick blood specimen was collected and tested at AFRIMS, as described below, for anti-HEV total Ig. Potential controls with evidence of prior HEV infection (AFRIMS recommended cutoff of≥20 WRAIR U/ml) were ultimately removed from the control pool, as circulating anti-HEV Ig is may be protective against future re-infection or hepatitis E illness.

Each candidate case and matched control was visited up to three times to maximize recruitment. Refusals were visited by supervisors to address any concerns about the risks or benefits of participation. All acute cases were visited by a study medical officer, who provided a thorough physical exam, supportive treatment as indicated, and referral for outpatient or inpatient care at the Matlab Hospital.

### Specimen Collection

A fingerstick blood specimen (∼350 µl) was collected from consenting and eligible cases and controls using a specialized capillary collection and separation microtube system (Safe-T-Fill, RAM Scientific, Needham, MA, USA) following procedures established by the manufacturer. Within 4 hours, specimens were centrifuged at 4000 g for 10 minutes and the supernatant serum was aliquoted testing and archival. Potential specimens transferred to Dhaka arrived within∼8 hours of shipment, at which time they were frozen at−20°C or processed immediately. Remaining aliquots of case and control sera were stored at−20°C until shipped on solid CO_2_ (dry ice) to AFRIMS for confirmatory testing.

### Laboratory Analysis

#### First Tier “Preliminary” Screening

Before subjecting candidate cases to an extensive interview process and recruiting matched controls, a small aliquot of serum was tested by the Molecular and Serodiagnostic Unit of the Laboratory Sciences Division (ICDDR,B) using a commercial anti-HEV IgM EIA (test kit ref. 1056) from Medical Biological Service (M.B.S. S.R.L, Milan, Italy) which uses antigens from conserved regions of HEV. This first tier screening was necessary for the expedient enrollment of candidate cases and controls, before final confirmation from AFRIMS was available.

Previous studies have shown that antibodies to HAV are ubiquitous in this population by the age of 10 [35]. As such, only children under 10 were also tested for anti-HAV IgM in the event that captured clinical disease was caused by HAV. A commercial EIA for anti-HAV IgM (DiaSorine, Italy) was used, according to the manufacturer's protocol.

#### Second Tier “Confirmatory” Screening

For the definitive confirmation of case or control status, specimens were tested using commercial and in-house EIAs for hepatitis A, B, and E antibodies by the AFRIMS (Bangkok, Thailand) Virology laboratory. Although commercial anti-HEV assays were available, the appropriateness of their use for epidemiologic studies is often questioned [2], [36]. The Walter Reed Army Institute of Research (WRAIR, Bethesda, MD, USA) has developed both quantitative anti-HEV IgM and anti-HEV total Ig EIAs which are recognized as a highly sensitive and specific to identify HEV infections in populations [37], [38]. Cases were tested for anti-hepatitis E virus IgM (anti-HEV IgM), anti-hepatitis E virus total Ig (anti-HEV Ig), and anti-hepatitis B virus core antigen IgM (anti-HBc IgM). Cases under 10 years old were also tested for anti-hepatitis A IgM (anti-HAV IgM) to identify possible cases of co-infection. As previous studies in this population demonstrated a low prevalence of HCV infections, anti-HCV was not tested [34]. Controls were tested for anti-HEV total Ig. Initially reactive specimens were tested in duplicate. Positive and negative controls were included in every EIA run.

The AFRIMS in house anti-HEV assays use a quantitative sandwich approach to capture and label human IgM and Ig antibodies to recombinant HEV open reading frame (ORF) 2 proteins (rHEV) of the Pakistani strain expressed in the baculovirus system. The photometric result is compared to positive controls from a positive reference pool of Nepali HEV patients, to report antibody concentrations in WRAIR units/milliliter (WRAIR U/ml). For total Ig, a cutoff of≥20 U/ml is used by WRAIR to classify subjects as ‘definite past infections’, and>1000 U/ml as definite acute HEV infection. For anti-HEV IgM, WRAIR uses≥100 U/ml as a diagnostic cutoff for acute HEV infection [39]. The protocol for these assays are described in detail elsewhere [37], [38].

### Statistical Methods

Data were entered using customized data entry screens, with range and consistency checks built in using Visual FoxPro (Microsoft Corp., Seattle, WA, USA). Statistical analyses were performed using Stata Version 9.0 for Windows (Stata Corporation, College Station, TX). A *p*-value of<0.05 was considered statistically significant for all analyses.

The performance of the Medical Biologic Service (MBS) anti-HEV IgM EIA was evaluated (sensitivity, specificity, positive and negative predictive values) against the AFRIMS IgM EIA (Table 2). Two case definitions were used to identify potential risk factors for acute hepatitis E. The first, more conservative definition, considered only second-tier, AFRIMS-confirmed, anti-HEV IgM seropositive cases. The second, more liberal, case definition uses anti-HEV seropositive cases detected by either MBS or AFRIMS methods. A study recruitment diagram was created to illustrate the enrollment of cases and controls (Figure 2).

**Table 2:** Correlation between AFRIMS and commercial anti-HEV IgM Assay.

		AFRIMS*			
		+	−	*Total*	
MBS**	+	19	24	*43*	*PPV: 44.2%*
	−	6	220	*226*	*NPV: 97.3%*
	*Total*	*25*	*244*	*269*	
		*Sensitivity*	*Specificity*	*88.5% Agreement*	
		*76.0%*	*90.2%*	*Kappa: 0.50*	

* AFRIMS in-house anti-HEV IgM Immunoassay as described in Myint 2006.

** Anti-HEV IgM Enzyme Immunoassay 1056, Medical Biological Service (MBS), Milano, Italy.

Case and control groups were compared descriptively. Demographic variables were explored by measures of central tendency and spread. The mean values of continuous variables were compared between groups by paired t-tests, where appropriate or non-parametric comparison of medians if necessary. After ineligible individuals were removed from the control pool, the 1∶4 intended case-control ratio was disrupted, leading to varying numbers of controls per case. Therefore, to estimate the significance and strength of association between sporadic hepatitis E and putative risk factors, accounting for variable case: control ratios, univariate conditional (fixed-effects) logistic regression was used to extend McNemar's test to multiple, differing numbers of controls per case. The inverse natural log of the coefficient was taken to derive the estimated odds ratio and their associated 95% confidence intervals.

Demographic variables assessed included age, gender, religion, marital status, household size (divided into tertiles), type of primary and secondary employment, nutritional status (proxied by mid-upper arm circumference (MUAC)), education, income category (lower than median, median, higher than median), and type of household construction. Household construction was proxied using a household score, summing ordinal values issued to the materials used to build the floor, walls and roof of the primary living quarters, in order of increasing expense and durability (e.g. wall quality could range from thatch/sticks, bamboo, wood to tin, to bricks/cement). The analysis of marital status and education associated with hepatitis E excluded persons aged 15 or less for marriage, and persons aged less than 7 years for education. MUAC was only compared among persons over 15 years old, as cutoffs used as indicators of malnutrition vary for growing children.

Exposures and behaviors assessed ranged from ownership of livestock and poultry, land use, ‘social empowerment’ group membership, travel to urban areas, and food consumption outside the home to contact with generally sick or specifically hepatitis patients, and injection or transfusion exposures. We also asked about individual and household sanitary practices (toilet type, distance from living quarters, toilet cleaning frequency, handwashing practices after toilet use and before eating), water use for drinking, cooking and washing, storage and/or filtration of water. Finally, given interest in a potential zoonotic component to HEV transmission, we assessed a range of potential exposures to livestock (feeding, milking, bathing or slaughtering) and creating “exposure” scores reflecting the number of activities an individual engaged in with a particular type of domestic animal. We explored the use of animal dung at the household level by creating a “dung exposure” score, reflecting the myriad ways in which a given household used animal dung (e.g. fuel, construction/repair, cleaning, and fertilizer).

Based on the results of univariate analyses, a model was developed using multivariate conditional logistic regression, controlling for possible confounders such as gender and indicators of socioeconomic status. Categorical dummy variables were used for non-linear, nominal covariates or where effect modification was suspected. Two modeling exercises were performed, based on the conservative and liberal case definitions. Due to the low numbers in any given cell using the conservative case definition, covariates of potential value, significant at the p<0.25 level in univariate analyses were added manually, and any variables retaining significance at the 25% level were preserved in the final model. Improved explanatory fit was grossly assessed by the pseudo-R^2^ estimator provided by Stata, with 1.0 representing best fit. When using the liberal case definition, a more stringent cutoff of 15% was used for inclusion in the final manually-fitted model.

A software-driven stepwise modeling approach was also used, including any variable significant at the 25% level during univariate analysis. In an exploratory study, such as this one, the use of too stringent pre-selection criteria can be counterproductive, as case cell sizes may be small for a particular exposure. Remaining variables were added in both a forward and backward stepwise manner, excluding or including variables above or below a 20% (p<0.2) significance level, respectively. This cutoff was lowered to 10% (p<0.1) when using the liberal case definition. The step-by-step addition of variables to the multivariate model identified the variables most strongly associated with the outcome (incident hepatitis E disease) while simultaneously adjusting for confounders. A bootstrap method was used to estimate the robustness of the final automatic model with 50 replications.

The final model provided an exploration of possible associated risk factors with incident, sporadic hepatitis E disease, allowing the calculation of an odds ratio for each significant variable, adjusting for the other covariates in the model. However, as an *exploratory* case-control study and not an inference-based or hypothesis-testing study, the final model is intended to inform future investigations of fixed hypotheses, indicating putative directions of association, without necessarily reflecting accurate magnitudes.

## Results

### Surveillance

The entire population of Matlab Block C, comprising 20 villages and an estimated population of 23,500 (mid-year 2003), was under hepatitis surveillance by 13 CHRWs. Between August 22, 2004 and May 31, 2006, 22 consecutive surveillance rounds were carried out, each lasting one month. This accounted for over 286 person-months of CHRW surveillance, covering over 43,000 person-years of exposure time.

### Numbers of Cases

Over this period, 279 potential cases of hepatitis E illness were identified by qualifying Illness Score and time since onset. The median illness score among this group was 6 (n = 143, 51.3%), with only the top 12.5% of scores reaching the maximum possible 8 (n = 35). The median time of onset was 14 days (mean 15.1±7.3 days) with only 11.1% being met within a week of symptom onset. Only 9 individuals remained not met, despite at least 3 attempts to enroll them, and none of the potential cases refused enrollment (See Figure 2).

The first-tier screening of the 269 candidate cases identified 43 anti-HEV positive cases who were subsequently interviewed. Of 269 specimens sent to AFRIMS for testing, 25 had an anti-HEV IgM≥100 U/ml and 4 had an anti-HEV total Ig>1000, for a total of 29 AFRIMS positive cases. Eight individuals originally classified as anti-HEV negative by first-tier screening were identified as positive by the second-tier AFRIMS screening. However, only 3 of those 8 individuals were able to be interviewed.

Table 2 compares the two IgM assays. The 4 individuals identified by AFRIMS using total Ig, while identified as cases, were analyzed as IgM negative in Table 2. Using the AFRIMS assay as the gold standard suggests a low positive predictive value of the MBS assay, at 43.2%. The kappa score (0.50) between the two assays suggests only a moderate level of agreement. This motivated the use of two case definitions in the analyses, one more conservative, using only AFRIMS-confirmed cases, and one more liberal, using either MBS or AFRIMS-confirmed anti-HEV seropositive cases. Inter-assay variability has been a contentious issue in HEV research, explaining our insistence on using both a commercial, clinical assay and an in-house research assay.

### Etiology of hepatitis illness

Of the 270 cases of suspected acute hepatitis illness identified, 49 (18.2%) of individuals were anti-HEV IgM positive by at least one EIA. As specimen volumes allowed, specimens were also tested for anti-HBc and anti-HAV IgM. Among 248 tested for both these antibodies, 18 (7.3%) were found to have acute antibodies to HBc, and 33 (13.3%) to HAV. This left 174 (64.4%) of the cases of acute hepatitis-like illness with an unexplained etiology, possibly representing non-specific morbidity, or over-reporting of symptoms due to expectations of treatment or perceived benefit of enrollment. Other etiologies could not be explored due to the low sample volumes remaining after initial testing.

### Number of Controls

A total of 268 potential controls were pre-selected for screening to match to candidate cases. Three were not met after 3 visits, 47 had moved temporarily and 4 others permanently, out of the Matlab area. Of the 214 visited, only 6 (2.8%) refused to participate. The 208 remaining potential controls were screened for eligibility (disqualified by a previous history of jaundice, and if no history of jaundice, a lifetime hepatitis illness score>4); this process disqualified 20 additional individuals. Four were dropped from the final analysis as their case counterpart (n = 1) moved out of the study area after initial enrollment.184 controls were finally identified, enrolled, interviewed and tested for antibodies to HEV by the AFRIMS research assay (Figure 2).

None of the 184 age-matched controls had an anti-HEV IgM titer greater than the AFRIMS recommended cutoff of 100 WRAIR U/ml for acute infections. The median titer was 7.2 WRAIR U/ml, ranging from 0.1 to 60.4 WRAIR U/ml (mean: 10.4±10.9 WRAIR U/ml). To exclude individuals with historical, subclinical HEV infections, the anti-HEV total Ig titers of the remaining 184 controls was assessed. The control pool revealed a median anti-HEV Ig level of 8.5 WRAIR U/ml (mean 28.6±62.6 U/ml). Using the recommended cutoff of≥20 WRAIR U/ml as indicative of previous HEV infection, 50 (27.2%) controls were removed, creating a completely seronaïve control pool. This exclusion led to a final case:control ratio of 2.9, with a final 134 controls for 46 cases.

### Liberal Case Definition

#### Dual Infections with HAV or HBV

Nine cases under 10 years old were identified under the liberal case definition. Three of these cases showed evidence of infection by HAV as well. Only two of the 46 cases were also identified as acute HBV infections by the Murex Abbott assay, one of whom was under 10 years of age. None of the HEV cases identified using the conservative case definition were found to be co-infected with HAV or HBV.

#### Characteristics of cases and controls

Cases were a median of 18.8 years old (mean 23.1±14.7). Thirty percent of cases were aged 15 or less, with 8 under age 10. As expected, cases were not significantly different in age from controls, confirming that age-matching of the groups was retained even after the withdrawal of non-eligible controls.

Univariate analyses of demographic characteristics and exposures of interest are displayed in two tables (Tables 3 & 4). Female gender was significantly protective of hepatitis E (odds ratio (OR) 0.38 95%CI: 0.18–0.76). Marital status, household size, or blood group were not significantly associated with illness. Common proxies of socioeconomic status (SES) such as education, household income, and house construction were also not associated with hepatitis E in this study.

**Table 3:** Demographic characteristics of acute hepatitis E disease patients and age-matched controls.

	Conservative Case Definition			Liberal Case Definition		
	Cases	Controls	Odds Ratio	Cases	Controls	Odds Ratio
	(N = 24)	(N = 68)	(95% CI)	(N = 46)	(N = 134)	(95% CI)
Demographic Characteristics	No. (%)	No. (%)		No. (%)	No. (%)	
**Age**						
≤15 years	6 (25.0)	23 (33.8)	-	14 (30.4)	53 (39.6)	-
16–30 years	14 (58.3)	40 (58.8)	-	19 (41.3)	52 (38.8)	-
>30 years	4 (16.7)	5 (7.4)	-	13 (28.3)	29 (21.6)	-
**Gender**						
Male	17 (70.8)	27 (39.7)	1.0	31 (67.4)	57 (42.5)	1.0
Female *	7 (29.2)	41 (60.3)	0.34 (0.13–0.93)	15 (32.6)	77 (57.5)	0.38 (0.18–0.76)
**Religion**						
Muslim	14 (58.3)	37 (54.4)	1.0	33 (71.7)	84 (62.7)	1.0
Hindu	10 (41.6)	31 (45.6)	0.76 (0.28–2.03)	13 (28.3)	50 (37.3)	0.58 (0.26–1.27)
**Marital Status** **						
Single (≤15 y)	6 (25.0)	23 (33.8)	-	14 (30.4)	53 (39.6)	-
Single	11 (45.8)	30 (44.1)	1.0	15 (32.6)	40 (29.9)	1.0
Married	7 (29.2)	15 (22.1)	0.37 (0.04–3.70)	17 (37.0)	39 (29.1)	0.39 (0.08–1.97)
**Household Size**						
≤4	7 (29.2)	15 (22.1)	1.0	16 (34.8)	34 (25.4)	1.0
5–6	12 (50.0)	32 (47.1)	1.04 (0.25–4.41)	19 (41.3)	56 (41.8)	0.74 (0.29–1.84)
7+	5 (20.8)	21 (30.9)	0.51 (0.12–2.15)	11 (23.9)	44 (32.8)	0.51 (0.20–1.30)
**Primary Employment** †						
None/Housework	2 (9.5)	14 (20.9)	1.0	4 (9.8)	35 (26.7)	1.0
Farming/Fishing/Labor	4 (19.1)	5 (7.5)	3.69 (0.53–25.5)	9 (22.0)	9 (6.9)	8.22 (1.67–40.49)
Own business/Rickshaw	3 (14.3)	4 (6.0)	0.99 (0.06–16.99)	6 (14.6)	10 (7.6)	5.11 (0.74–35.16)
Office-based service/Teaching	2 (9.5)	1 (1.5)	8.53 (0.48–151.78)	2 (4.9)	1 (0.8)	17.60 (0.92–337.31)
Student	9 (42.9)	38 (56.7)	1.88 (0.28–12.43)	17 (41.5)	61 (46.6)	6.23 (0.95–40.65)
Minor Child	1 (4.8)	5 (7.5)	1.15 (0.03–41.76)	3 (7.3)	15 (11.5)	2.45 (0.10–60.95)
**Gross Nutritional Status** ¶						
MUAC≥22.5 cm	12 (66.7)	23 (51.1)	1.0	21 (65.6)	48 (59.3)	1.0
MUAC<22.5 cm	6 (33.3)	22 (48.9)	0.64 (0.16–2.51)	11 (34.4)	33 (40.7)	0.95 (0.37–2.46)
**Education** ‡						
None	2 (8.3)	8 (11.8)	1.0	6 (13.0)	28 (20.9)	1.0
Class 1–5	12 (50.0)	26 (38.2)	2.17 (0.35–13.34)	21 (45.7)	44 (32.8)	2.65 (0.89–7.89)
Class 6+	10 (41.7)	34 (50.0)	1.16 (0.20–6.93)	19 (41.3)	62 (46.3)	1.46 (0.49–4.39)
**Monthly Household Income**						
< Taka 3000 (<US$45)	2 (8.3)	7 (10.3)	1.0	7 (15.2)	17 (12.7)	1.0
Taka 3000–4999 (US$45–75)	16 (66.7)	33 (48.5)	1.39 (0.26–7.37)	25 (54.4)	59 (44.0)	0.98 (0.36–2.68)
> Taka 5000 (>US$ 75)	6 (25.0)	28 (41.2)	0.64 (0.11–3.75)	14 (30.4)	58 (43.3)	0.55 (0.19–1.59)
**Household Construction** §						
Impermanent (Thatch, mud) reeds)	4 (16.7)	9 (13.2)	-	7 (15.2)	16 (11.9)	-
Semi-permanent (Tin walls)	20 (83.3)	56 (82.4)	-	39 (84.8)	113 (84.3)	-
Permanent (Cement walls)	0 (0.0)	3 (4.4)	0.58 (0.28–1.22)	0 (0.0)	5 (3.7)	0.66 (0.39–1.12)

NB. Conservative case definition includes only individuals found to be anti-HEV IgM seropositive by the AFRIMS assay. The liberal case definition includes individuals found positive by either AFRIMS or the MBS assay.

* p<0.05, Univariate conditional (fixed effects) logistic regression comparing cases and controls.

** Comparison of marital status excluded individuals ineligible for marriage as an exposure as age≤15, Divorced/Widowed not shown (n = 2).

† “Don't Know” responses excluded, each category tested against “None/Housework”, adjusting for other categories of employment in a multivariate conditional logistic regression model.

¶ Poor nutritional status defined by Mid-upper Arm Circumference (MUAC)<22.5. MUAC comparison was restricted to participants>15 y.o.

‡ Comparison of education excluded individuals ineligible for schooling as an exposure as age<7.

§ A household score was created based on the sum of ordinal values issued for the type of roof, floor and wall construction. The OR was calculated for every one unit increase in household score (p>0.05, NS).

**Table 4:** Exposure characteristics of acute hepatitis E patients and age-matched controls.

	Conservative Case Definition			Liberal Case Definition		
	Cases	Controls	Odds Ratio (CLR)	Cases	Controls	Odds Ratio (CLR)
	(N = 24)	(N = 68)	(95% CI)	(N = 46)	(N = 134)	(95% CI)
Exposure Characteristics	No. (%)	No (%)		No. (%)	No (%)	
**Primary Employment Location**						
Indoors	11 (45.8)	40 (58.8)	1.0	15 (32.6)	69 (51.5)	1.0
Outdoors	13 (54.2)	28 (41.2)	1.63 (0.57–4.62)	31 (67.4)	65 (48.5)	3.17 (1.35–7.47)
**Livestock Ownership** *						
Cows (Any)	5 (20.8)	27 (39.7)	0.35 (0.10–1.15)	13 (28.3)	53 (39.6)	0.62 (0.29–1.31)
Goats (Any)	5 (20.8)	19 (27.9)	0.61 (0.18–2.12)	13 (28.3)	30 (22.4)	1.42 (0.63–3.24)
Chickens (Any)	11 (45.8)	38 (55.9)	0.52 (0.18–1.47)	26 (56.5)	72 (53.7)	1.05 (0.53–2.07)
Ducks (Any)	14 (58.3)	34 (50.0)	1.17 (0.45–3.05)	29 (63.0)	65 (48.5)	1.58 (0.79–3.16)
**Recent** ** **travel to town/city**						
None	6 (25.0)	45 (66.2)	1.0	20 (43.5)	90 (67.2)	1.0
Any §	18 (75.0)	23 (33.8)	10.23 (2.17–48.15)	26 (56.5)	44 (32.8)	2.37 (1.15–4.87)
**“Eating Out” frequency** †						
Never	17 (70.8)	57 (83.8)	1.0	28 (60.9)	106 (79.1)	1.0
<7 times/wk	5 (20.8)	6 (8.8)	2.98 (0.77–11.56)	12 (26.1)	18 (13.4)	3.15 (1.16–8.52)
7+ times/wk	2 (8.3)	5 (7.4)	0.63 (0.07–5.51)	6 (13.0)	10 (7.5)	1.80 (0.58–5.56)
**Recent “Jaundice” contact** **, ‡						
None	17 (73.9)	64 (97.0)	1.0	34 (75.6)	127 (97.0)	1.0
Any §	6 (26.1)	2 (3.0)	10.83 (2.18–53.79)	11 (24.4)	4 (3.1)	9.63 (3.06–30.37)
**Recent Traditional Healer Visit** **						
None	5 (20.8)	59 (86.8)	1.0	12 (26.1)	118 (88.1)	1.0
Any §, ‡‡	19 (79.2)	9 (13.2)	39.45 (5.18–300.24)	34 (73.9)	16 (11.9)	35.55 (8.42–150.05)
**Recent Injection** **						
None	16 (66.7)	55 (80.9)	1.0	29 (63.0)	98 (73.1)	1.0
Any	8 (33.3)	13 (19.1)	1.80 (0.55–5.87)	17 (37.0)	36 (26.9)	1.86 (0.79–4.35)
**Type of Household Latrine**						
Open/Hanging (Unsanitary)	16 (66.7)	30 (44.1)	1.0	25 (54.4)	56 (41.8)	1.0
Sealed/Slab (Sanitary) ||	8 (33.3)	38 (55.9)	0.28 (0.09–0.90)	21 (45.7)	78 (58.2)	0.54 (0.26–1.13)
**Drinking Water Source** ‡						
Tubewell	23 (100.0)	58 (85.3)	-	42 (93.3)	121 (90.3)	1.0
Pond	0	2 (2.9)	-	1 (2.2)	3 (2.2)	1.22 (0.10–14.4)
River	0	8 (11.8)	-	9 (6.7)	2 (4.4)	0.69 (0.14–3.46)
**Cooking Water Source**						
Tubewell	2 (8.7)	3 (4.4)	1.0	3 (6.7)	5 (3.7)	1.0
Pond	17 (73.9)	43 (63.2)	0.56 (0.09–3.41)	32 (71.1)	88 (65.7)	0.60 (0.14–2.53)
River	4 (17.4)	22 (32.4)	0.23 (0.03–1.98)	10 (22.2)	41 (30.6)	0.36 (0.07–1.89)
**Average Water Consumption** ††						
≤7 glasses per day	7 (29.2)	37 (54.4)	1.0	14 (30.4)	72 (53.7)	1.0
≥8 glasses per day	17 (70.8)	31 (45.6)	3.11 (0.92–10.55)	32 (69.6)	62 (46.3)	2.83 (1.19–6.74)

NB. Conservative case definition includes only individuals found to be anti-HEV IgM seropositive by the AFRIMS assay. The liberal case definition includes individuals found positive by either AFRIMS or the MBS assay.

* “None” is not shown, comparison of livestock ownership uses “None” as the reference category (OR = 1.0).

** Recent is defined as in the three months prior to the onset of illness/interview

† Defined as the number of times eating food prepared outside the home in an average week.

§ p<0.005, Univariate conditional (fixed effects) logistic regression (CLR) comparing cases and controls.

‡ “Don't know” (n<5) responses not shown/included in analysis.

|| p<0.05, Univariate conditional (fixed effects) logistic regression comparing cases and controls.

†† In an age category stratified analysis (≤15, 16–30,>30) only the≤15 group remained significantly at risk, likely due to low n's in each age category.

‡‡ This association likely reflects a local practice of seeking initial care from traditional “faith” healers early during hepatitis-like illnesses.

In the analysis of various employment categories compared to none/housework, a significant increased risk was seen for fishing/farming and outdoor labor professions (OR: 8.22, 95%CI:1.67–40.49). A second analysis of employment-related risks excluding the influence of persons aged<15 (who may be protected from common employment-specific exposures) estimated the association of infection with fishing/farming and outdoor labor at 13.8 (95%CI: 1.66–115.69). In this analysis, the association between hepatitis E and office-based employment was also significant (OR 29.07, 95%CI: 1.09–772.69); this result should be interpreted with caution due to small numbers in each case-control matched group.

Participants reporting their primary work environment as being “outdoors” were at an estimated 3-fold higher risk of hepatitis E than indoor working counterparts (OR 3.17, 95%CI: 1.35–7.47). Travel in the three months prior to illness to an urban area (town or city) for these village-dwelling cases carried a 2.37 (95%CI: 1.15–4.87) fold increased risk. Also in line with these results was a significant increased risk among individuals who ate food prepared outside the home between 1 and 6 times every week (OR 3.15, 95%CI: 1.16–8.52). However, those eating food prepared outside the home>7 times a week were not significantly associated with hepatitis E (albeit again, low numbers preclude any definitive conclusion from this data).

Contact with any sick person in the 3 months prior to illness was not a significant risk factor, but interestingly, reported contact over the same period with an individual suffering from yellow eyes or skin carried a∼10-fold increased risk of being a case (OR 9.63 (95%CI: 3.06–30.37). Subjects were also asked if they had visited a traditional healer (*Kabiraj/Ouja/Fakir*) in the three months prior to illness. This behavior was highly associated with hepatitis E, with an odds ratios of 35.55 (95%CI: 8.42–150.05) (Table 4).

Tubewells, which are generally considered pathogen-free in most situations, are nearly universally used as the source for drinking water in this community (91.1%,). Despite over 90% household tubewell ownership in this population, the use of “unsafe” water in both groups for cooking was ubiquitous, largely collected from a pond (n = 150, 64.4%) or river (n = 71, 30.5%). Drinking water, if not consumed immediately, was stored in covered aluminum containers (86.1%). These behaviors were not associated with hepatitis E illness in this dataset. Although 70.2% of 181 respondents reported drinking from a tubewell with “unsafe” arsenic levels, our data was not able to discriminate any association with acute hepatitis E.

Individuals consuming 8 or more glasses of water per day (about 70% of cases), had an OR of 2.83 (95%CI:1.19–6.74), compared to those consuming 7 or fewer glasses. (The median reported across the entire study population was 8 glasses per day.) However, this association may be confounded by age as younger children are less likely to consume large amounts of water. Stratifying this analysis by age category (≤15, 16–30,>30 - data not shown) resulted in low numbers per cell, but the positive association remained significant in individuals 15 or younger (OR 6.57, 95%CI: 1.28–33.59), with 19% of cases consuming over 8 glasses per day. For comparison, in the 16–30 age stratum, 63% of cases and in the>30 age stratum, 86%, of cases reported consuming over 8 glasses of water a day. Positive associations with water intake in the age-stratified analysis above age 15 were not statistically significant.

Using a sanitary latrine (sealed or slab) was not found to be significantly protective compared to an open pit or “hanging” latrine using this definition. Although sanitary toilets were more likely to be near the living rooms compared to unsanitary toilets, toilet location had little effect on the magnitude or significance of the protective association. Toilet cleaning involvement or frequency had no significant association with hepatitis E. Self-reported hand washing before eating was universal. Most respondents used only water, with only 10.4% using soap and water. Only 16.3% reported washing *both* hands before eating, a typical cultural practice across rural Bangladesh; this was, however, not different between cases and controls. Hand washing after defecation was also nearly universal (98.3%), with a variety of methods used, ranging from water only (40.0%), to soap and water (39.1%), and earth/ash with water (20.9%); only 68.5% of respondents reported washing both hands, also not different between cases and controls.

Livestock (cows, goats) and fowl (chickens, ducks) ownership was common in this rural community at 37%, 24%, 54% and 52%, respectively, among study participants. Exposure to these animals in terms of bathing, feeding or milking was minimal, and not associated in this data with illness. Contact with animal feces, namely cow dung, is common in rural Bangladesh; 87.0% of both cases and controls reported using dung, primarily for house repairing and maintenance (50.0%) and as cooking fuel (32.5%). Use of dung was not associated with hepatitis E. Recent studies have investigated whether pets or pests may serve as risks for the transmission of HEV [40]; in this study over 98% of cases and controls had seen a rat in or near their home in the month prior to illness. Domesticated dogs or cats were extremely rare in this population.

Nearly 85% of participants reported a pond in their homestead; given the reported use of pond water for cooking, we investigated the ownership and size of household ponds as well as the level of pond water in the month before illness, but no associations with hepatitis E disease were found. No association with recent flooding was evident in this study either. Also non-significant were microcredit or social-empowerment NGO membership, 3-month history of injection exposure, transfusions of blood, and the first level of health care sought by the individual during illness. Community ‘roaming’ barber use for shaving or haircutting was frequent (overall 79%), but showed no association with hepatitis E.

Interestingly, 29% of study participants, irrespective of case status, reported having had an injection in the previous 30 days. Excluding women who also reported injectable contraceptives in the past year (n = 5), this rate remained high among men (26.1%) and women (27.2%).

#### Multivariate Analysis

A larger number of cases (n = 46) were available for this analysis with more matched controls (n = 134). The results of both the forced “manual” model and the stepwise models are shown in Table 5. The variables selected (p<0.25 in the univariate analysis) for the liberal multivariate analyses included gender, level of education, employment category, income category, house construction score and size of household. Exposure variables selected were recent travel to a town or city, work outside the home, work outdoors, quantity of water normally consumed daily, source of cooking water, the habit of consuming food prepared outside the home, recent exposure to hepatitis-like illness, recent exposure to injections, level of treatment provider sought for normal illnesses, use of unsanitary latrine, practice of cleaning the latrine, ownership of and exposure to cows, chickens or ducks, and the diversity of dung use in the household.

**Table 5:** Multivariate conditional logistic regression model† of risk factors for HEV disease.

	Manual (forced) Model*			Stepwise Selected Model**		
Characteristic	OR	95% C.I.	p value	OR	95% C.I.	p value
Gender (0 = Male, 1 = Female) §,‡	0.30	0.07–1.20	0.088	-	-	-
Recent Exposure to “Jaundice” patient^‡^	63.50	8.07–499.50	<0.000	82.50	8.77–776.39	<0.000
Travel to town/city in past 3 months ^‡^	2.80	0.78–10.08	0.114	4.25	1.06–17.10	0.041
Unsanitary toilet use ^‡^	4.39	1.02–18.98	0.048	5.14	1.20–22.01	0.027
Work outside the home	19.36	1.39–269.75	0.027	19.80	1.89–207.96	0.013
Outdoor Work (Farming/Fishing/Labor)	4.17	0.73–23.78	0.108	8.63	1.33–56.09	0.024
Injection in the last 3 months	18.44	2.10–162.05	0.009	15.50	1.97–121.76	0.009
Household size (≤4, 5–6,≥7 members)	0.49	0.29–0.84	0.009	0.17	0.05–0.56	0.004
Household construction score (1–6)	-	-	-	0.35	0.13–0.98	0.045

† Only the models constructed using the liberal case definition are shown as the conservative case definition was extremely restricted by a low sample size. Variables also significant using the conservative model are those indicated above by an (^‡^).

* Model reflects explanatory variables of interest significant at the 25% level (p<0.25) in univariate conditional logistic regression, and retained at least 15% (p<0.15) significance in the multiple, adjusted model.

** Model selected by forward and reverse stepwise conditional logistic regression, including variables significant at the 25% level (p<0.25) in univariate analysis and retained in the final mode if significant at the 10% (p<0.10) level.

§ Gender was left out of the stepwise model as female gender was strongly inversely correlated with work outside the home or with performing outdoor work. Stratified analysis was not possible or appropriate due to extremely small numbers in each cell.

The manually-selected model was quite similar to the stepwise selected model, except with gender forced into the model. Female gender seemed to be protective (albeit not significantly) against hepatitis E illness (OR 0.30, 95%CI:0.07–1.20, p = 0.088). Despite the increase in sample size by the liberal case definition, it was still too small to perform extensive gender- or age-stratified analyses. We looked at certain key variables to see if there were clear correlations between gender and behavior (e.g. work outside the home, travel to town/city). Of the 15 female cases, 40% reported visiting a town or city compared to 64.5% among the 31 male cases, not statistically different (Fisher's exact test, p>0.2). However, when the gender association with hepatitis E illness was adjusted by covariates such as travel to town, employment outside the home and outdoor work, for which gender is likely a proxy, the significance of gender decreased dramatically (data not shown).

The same final fitted model was selected by both forward and backward stepwise conditional logistic regression. All exposures finally selected were significant at the 5% level (p<0.05), adjusted for the other variables. Due to the limited sample sizes and wide confidence intervals of the final estimates of association, we focus here on direction, not magnitudes, of association. Positive associations were for reported exposure to a hepatitis or jaundiced patient in the three months prior to illness (OR 82.50, 95%CI:8.77–776.39), employment outside the home (OR 19.80, 95%CI:1.89–207.96), and injection exposure in the three months before hepatitis E (OR 15.50, 95%CI: 1.97–121.76). Other variables associated with increased risk of hepatitis E were outdoor work (farming/fishing or manual labor) (OR 8.63, 95%CI:1.33–56.09), unsanitary toilet use in the household (OR 5.14, 95%CI 1.20–22.01) and travel to a town/city in the past three months (OR 4.25, 95%CI: 1.06–17.10). Possibly protective against hepatitis E illness were increases in household size category (OR 0.17, 95%CI: 0.05–0.56) and improvements in household construction score (OR 0.35, 95%CI: 0.13–0.98).

The results of the 50 replicate “bootstrap” tests of the final model showed increased standard errors for some covariates (3 month history of visit to “jaundiced” patient, outdoor work and history of recent injection), and suggest caution in the interpretation of this small sample of cases and controls.

### Conservative Case Definition

This alternate analysis included only 24 cases confirmed by AFRIMS with anti-HEV IgM≥100 WRAIR U/ml or anti-HEV total Ig>1000 WRAIR U/ml. Four age-matched controls without a lifetime history of jaundice were retained for each case, with subsequent elimination of 28 controls with anti-HEV total Ig≥20 WRAIR U/ml. The conservative analyses were conducted with 24 cases and 68 age-matched controls, a ratio of 2.83 controls per case.

#### Characteristics of cases and controls

The median age was 17.1 years (mean 20.9±11.5) with 25% under 15 years and only 2 cases under 10 years old (Table 3). As with the liberal definition, female gender was significantly associated with decreased risk of hepatitis E (OR: 0.34 95%CI: 0.13–0.93). However, the association of illness with fishing, farming and outdoor labor professions was no longer observed. Similarly, while an outdoor work environment had a 63% increased risk, this association was not significant. Recent travel to an urban area was associated with increased risk in illness (OR 10.23 95%CI: 2.17–48.15). Recent contact with a person displaying symptoms of jaundice remained a significant risk factor under this definition with an OR of 10.83 (95%CI: 2.18–53.79) along with a recent visit to a traditional healer (OR: 39.45 95%CI: 5.18–300.24) (Table 4).

All cases in this analysis reported use of tubewell water as their drinking water source; therefore, water source could not be statistically linked with risk of hepatitis E. Similar to the liberal case definition, the use of unsafe water for cooking was also common and not different between cases and controls. There was an over 3 fold increased risk of HE for individuals consuming 8 or more glasses of water per day, however, this association was no longer significant using this conservative case definition (Table 4). Interestingly, in this analysis, the use of sanitary latrines, as opposed to open pit or “hanging” latrines, was significantly associated with a decreased risk of illness (OR: 0.28, 95%CI: 0.09–0.90).

#### Multivariate Analysis

Unadjusted variables, significant at the 25% level (p<0.25) that were included manually and automatically (forward and backward stepwise) in a multivariate conditional logistic regression model included gender, employment category, house construction score and NGO membership. Exposure variables selected were recent travel to a town or city, work outside the home, fish pond size, level of water in the pond, unsanitary latrine use, ownership of and exposure to cows, chickens or ducks, recent exposure to a jaundiced person, level of treatment provider sought for normal illnesses, source of cooking water, source of drinking water, water storage behavior, quantity of water normally consumed daily and dung use in the household. The variables preserved by the final automatic model were recent visit to a town (OR 29.53, 95%CI: 2.58–337.72), unsanitary toilet use (OR 5.28, 95%CI: 0.89–31.57), dung use score (OR 0.25, 95%CI: 0.08–0.84), and 8 or more glasses of water consumed daily (OR 4.37, 95%CI: 0.65–29.14).

## Discussion

We recently reported a high population rate of HEV infection (near 60 per 1000 person-years), with a lower rate of accompanying disease in this population [5]. In contrast to neighboring Nepal and India, which experience large annual outbreaks of hepatitis E, there are few reports of outbreaks from Bangladesh [34], although clinical studies confirm the important burden of hepatitis E in this population [41], [42]. This study represents one of the first efforts to identify putative risk factors for sporadic HEV infection and illness, in this rural South Asian context.

The difficulties in conducting studies of sporadic HEV are obvious, given the need for large, population-based surveillance systems to identify incident hepatitis E in communities. In order to rapidly identify and enroll candidate cases, the use of a commercial anti-HEV assay was necessary; this choice has unfortunate caveats given the relatively lower performance of commercially available anti-HEV assays, including the clinical assay used in this study, when compared to reference laboratory in-house assays (Table 2). As an important number (n = 22) of candidate cases were not confirmed by our secondary screening (Figure 2), two case definitions were finally used to analyze this study data. As an exploratory venture to identify potential risk factors for sporadic HEV infection and disease, results under either case definition are presented, with caution against the over-interpretation of findings of association, due to low final numbers and potential for case misclassification and bias. This was an exploratory study designed to identify as many possible risk factors for incident hepatitis E as possible. Therefore, we chose to use strict exclusion criteria in the identification of controls. We also chose not to adjust the p-values for multiple comparisons so that potential risk factors would not be eliminated. The associations seen in this study have limited interpretability due to these assumptions and the small sample size of cases; however, these results are intended to be used as a guide for the design of future studies. Another important limitation is the unblinded interviews of the cases and controls which may contribute to several of the differences seen between cases and controls such as recent contact with a jaundiced patient or a traditional healer. This is unlikely to have had a large effect on our results as highly structured interviews were conducted for both the cases and controls. Additionally, these risk factors were not previously thought to play a major role in the spread of hepatitis E and therefore, it is unlikely, that interviewees would have biased the participants' answers.

### Low burden of hepatitis E disease

This rural Bangladeshi population is, by many measures, one in which enteric infections can flourish. The level of sanitation, although improving, remains low, with just under half (44%) of the subjects still using “open” or pit latrines. This risk is compounded by the prevalent use of unsafe pond and river water for cooking, washing and bathing. Widespread animal husbandry results in increased exposures to zoonotic infections. Physical contact with cow dung is ubiquitous as a source of fuel, fertilizer and for construction. It is clear why other enteric pathogens, such as hepatitis A, infect most individuals in rural Bangladesh early in life [41].

Over 22 months of surveillance, no outbreaks of hepatitis E were identified in this rural, riverine population of over 23,000. An underlying landscape of non-specific morbidity led to the identification of 279 candidate cases, only 35% of which could be etiologically specified as HEV, HAV or HBV infections. Up to 46 individuals were identified as acutely infected hepatitis E illness cases, by either an AFRIMS in-house or MBS commercial EIA. Of the 188 “healthy” controls selected as sero-naïve controls,∼27% were finally excluded due to antibody titer evidence of likely prior infection. This low to moderate prevalence of anti-HEV among ‘healthy’ individuals is consistent with a recent prior study in this same population [34].

Cross-sectional and population based studies in Egypt, where HEV outbreaks are also not seen despite high anti-HEV seroprevalence and infection rates, suggest that strain-specific differences in virulence and infectivity may influence the manifestation of hepatitis E as outbreaks or sporadic cases [40], [43], even within a common genotype. Alternatively, the absence of large outbreaks or greater numbers of sporadic cases may reflect an insufficient environmental exposure to HEV to cause clinical disease. Although HEV may be pervasive throughout the Matlab community, the level of exposure necessary to cause disease may have been rare during the course of the study. Certain risk factors ranging from viral to host characteristics and concomitant environmental cofactors may directly increase the level of individual exposure, which then tips the balance towards overt clinical illness. Host micronutrient status and immunocompetence was not measured in this study, but could certainly be important in the response to HEV infection, whether subclinical, mild or severe.

Individuals may maintain low levels of anti-HEV from infections early in life, where present titers have fallen below the cutoffs used for prior infection. This may result in an underestimation of actual anti-HEV exposure in this population. Recent exposures to HEV may, in such individuals, result in anamnestic responses to re-infection without clinical illness [43]. The phenomenon of HEV infection without an antibody response (although not usual in immunocompetent individuals) has also been previously described [31] and may result in the underreporting or failure to capture incident HEV infections/disease. By design, this study compared cases to serologically naïve age-matched individuals, attempting to ensure that controls were at a theoretically equal risk of infection as the cases. Other environmental exposures which affect hepatic integrity, such as aflatoxin, might be involved in increasing the likelihood of clinical progression in infected persons.

### Age, Multiple Infection and Gender

Depending on the case definition, between 25-30% of the acute HEV cases identified in this study were under 15 years old. Much has been published on the paucity of pediatric HEV infections in this region, as gleaned from the sero-epidemiology of outbreak and hospital-based data [4], [44]. Among the 9 cases under 10 years of age, three (33%) were co-infected by HAV, and one by HBV. The increase in severity and manifestation of clinical illness in children co-infected by multiple hepatotropic viruses has been previously described [45], [46]. In an environment where multiple enterically transmitted viruses circulate, the increased likelihood of co-infection in susceptible young children is not surprising. Also as expected, 70% of the cases identified were in their second or third decade of life, supporting the seroepidemiologic evidence on delayed HEV infection from this region [47], [48].

Many early studies of HEV focused on the landmark characteristic of increased morbidity and mortality among pregnant women, especially in the third trimester [1], [49], [50]. Initial speculation about gender-related differences in susceptibility to infection has been replaced by the understanding that pregnancy seems to exacerbate HEV pathogenesis, rather than increasing the likelihood of infection itself [2], [51]. Studies in conservative communities in South Asia, where social restrictions on women's movement exist, have suggested a protective effect of female gender on HEV infection [15]. This study, too, suggested that female gender was protective against hepatitis E, possibly attributable to societal restrictions on movement, keeping women from work outside the home or frequent travel to urban areas [52].

### Demographic predictors

Sample size estimates were based on disease rates similar to those seen in India and Nepal, however, we observed a lower number of cases than expected. As a result, few differences in demographic characteristics were detectable in this study including no differences in indicators of SES, except the suggestion of increased risk to individuals employed outside the home, notably in professions with significant outdoor activity. These types of activity predispose individuals to resort to unsafe water consumption, whereas in the household, microbiologically “safe” tubewell water is more readily available.

### Behavioral predictors

Similarly, significant univariate predictors of hepatitis E were characteristics associated with exposures outside the home. In addition to “outdoor” job activities, consuming food cooked outside the home was associated with a three-fold increased risk of hepatitis E illness in one analysis. Cases were significantly more likely to report travel to a town or city (irrespective of gender) in the three months prior to illness. In the univariate analysis there was a consistent increase in the likelihood of cases reporting contact with a “jaundice” or “hepatitis-illness” patient in the three months prior to illness. Some of this association may be due to differential recall bias, as cases seek to explain their illness. However, epidemiologic investigations of a recent massive outbreak in Uganda suggested a strong role of person-to-person transmission in sustaining the epidemic [53]. In most outbreaks, secondary transmission has not been reported, as reflected in∼2% secondary attack rates and low intra-familial transmission reported in several historical, large outbreaks [54], [55]. However, this association deserves careful future assessment as a visit to a “jaundiced case” may expose a susceptible subject to contaminated drinking water or food, as fecal shedding of HEV has been well documented.

In univariate analyses, cases were significantly less likely to use sanitary latrines in their household. The use of unsanitary latrines increased the risk of hepatitis E (95%CI: 1.11-11.49). Open or “hanging” latrines allow human feces to be washed into nearby ponds and rivers, from which water may be collected for washing, bathing, and even cooking. Studies from Nepal and India have shown that HEV-infected individuals continue to be viremic and shed virus in stool for several weeks, even in the absence of antibody evidence of infection [31], [56]. The use of these “unsafe” water sources was not associated in this study with increased risk of illness, in contrast to findings from Indonesia and Vietnam [57], [58]. This study did document, as seen elsewhere in South Asia, the widespread use of these sources for cooking, bathing and washing [57], [59]. These behaviors, in combination with the widespread use of unsanitary latrines and the phenomenon of extended fecal shedding could work to maintain HEV endemicity, even if only through ongoing subclinical infections.

The consistent risk of hepatitis E among individuals (across all age categories) who reported consuming 8 or more glasses of water daily, although reduced in significance in an age-stratified analysis, is interesting. As mentioned earlier, the positive risk remained significant in the lowest age category of 15 or younger (OR 6.57, 95%CI 1.28–33.59) which raises the question of whether a critical dose of HEV is needed to cause clinical disease. Despite the ubiquitous environmental presence of HEV, perhaps only few individuals occasionally ingest sufficient virus to result in disease, compared to a larger number who are exposed and experience subclinical infection. As suggested by Clayson and colleagues, this cycle of continued inapparent HEV infections and shedding may maintain the virus in communities, even in the absence of large epidemics [4].

An association was found between hepatitis E and a visit to a village “Traditional Healer” in the three months prior to illness. Although it is possible that their practices (a mixture of herbalism, shamanism, and spiritual healing) may expose subjects to contaminated food or water, this association likely represents a reverse causal pathway. Traditional healers (*Kabiraj*) are often the first line of treatment for many illnesses in rural Bangladesh, and qualitative data from this population [33] suggests that as allopathic treatment for acute viral hepatitis is limited, traditional healers are sought for a wide range of alleged cures for hepatitis, soon after classic prodromal symptoms appear.

Modeling exposures to predict individual risks for sporadic hepatitis E was difficult, primarily due to a lower-than-expected number of cases. As a result, the interpretation of this study's data should focus on the direction of associations, and not necessarily their magnitude. The characteristics which remained in the final model, irrespective of case definition or method of variable selection, represent risk factors consistent with those predicted from sero-epidemiologic cross-sectional studies and outbreaks in this region. Poor household sanitation remained positively associated with hepatitis E, although improvements in household construction may impact on overall cleanliness, and decrease the risk of infection. A complex of exposures “outside the home” remained in the model, ranging from external employment and outdoor work (farming/fishing/labor), to travel to urban areas. Each of these behaviors potentially increases the risk of consuming unsafe food or water.

The persistence in the models of acute hepatitis E and reported contact with a jaundiced individual in the months prior to illness is difficult to explain and deserves closer investigation under a sporadic transmission paradigm. Increased household size seemed to be protective against illness, an association which might reflect an increased risk of early subclinical HEV exposure and subsequent “family” immunity to illness. Family size was not significantly associated with income or education, making it difficult to explain as a proxy for improved socioeconomic status. This association is opposite from the increased risk shown for HAV infection in larger families in India [60].

Finally, a positive association emerged in the multivariate model of reported exposure to injections (irrespective of gender) in the months preceding illness. This suggestion is perplexing, given the primary mode of HEV (GT 1) transmission in outbreaks has been fecal-oral through a waterborne route. However, there are suggestions in the literature that HEV can be transmitted parenterally [61], [62]. This route of possible HEV infection could be investigated further in conjunction with further exploration of an unexpectedly high prevalence of injection use in an otherwise “healthy” rural Bangladesh population.

### Zoonotic associations

A substantial literature has developed around zoonotic HEV infections in swine, deer and domesticated livestock in both endemic and non-endemic countries [63]–[65]. Although these infections have been shown to be of different genotypes (GT 3, 4) than that which is normally found in south Asian human cases (GT 1), cases of animal to human infection by GT 3 and 4 have been clearly documented [3], [63], [66]. Domestic pigs in India and Nepal were found to be infected with HEV (GT 4) early in life [67], [68]. In addition to pigs, a 2001 survey of different Indian animal species suggested high anti-HEV prevalence in cattle and even rodents (*Bandicota bengalensis*) [68], two ubiquitous exposures in this Bangladesh population under study.

As a predominantly Muslim community, pigs were not reported in any of the households studied in Matlab. We were also unable to demonstrate any significant association between other livestock, or with individual involvement in specific practices of feeding, washing or sacrificing animals. We were also unable to find any association between level of animal dung use in the household and disease. Rodents were reported by nearly all respondents, making it difficult to assess any role of these pests in the spread of HEV. It is, however, likely that most of the human HE seen in this population is not due to zoonotic genotypes, but rather caused by GT 1, as has been repeatedly shown in clinical studies [67].

There are certain population subgroups in Bangladesh (aboriginal tribes, and other non-Muslim minorities) that do raise and consume pigs. Further studies may elect to describe the persistence and protective efficacy of HEV antibodies, genotype variability and the zoonotic epidemiology of HEV in domestic livestock in rural Bangladesh; studies of the aforementioned subgroups at potentially increased risk for hepatitis E from swine reservoirs would also be useful [69].

## Conclusions

Sporadic hepatitis E remains difficult to explain, although this study suggests that HEV, ubiquitous in the environment, may be the source of continuous subclinical infection, causing disease in younger individuals when co-infections with other viruses occur, and in older adults (or also in children) when substantial virus ingestion occurs. These exposures that lead to illness may take place when individuals leave the relative “safety” of their home environment, and are exposed to unsafe water or food. Low levels of household sanitation may also increase the risk of infection. Further study is needed to clarify the possible parenteral and intrafamilial risks of HEV transmission, as well as continued investigation of the role, if any, which animals have in maintaining HEV in populations dominated by HEV GT1 infections.

This data provides future directions for the study of sporadic HEV, a better understanding of which will help us target high-risk and vulnerable groups for vaccine intervention. It seems likely that classical measures of improving community sanitation and lowering individual risk (avoiding potentially contaminated food or water from an outbreak or single sick individual) may also be effective in reducing HEV transmission and disease.
